# Evaluation of TGF-Beta 2 and VEGF*α* Gene Expression Levels in Epiretinal Membranes and Internal Limiting Membranes in the Course of Retinal Detachments, Proliferative Diabetic Retinopathy, Macular Holes, and Idiopathic Epiretinal Membranes

**DOI:** 10.1155/2018/8293452

**Published:** 2018-04-23

**Authors:** Joanna Stafiej, Karolina Kaźmierczak, Katarzyna Linkowska, Paweł Żuchowski, Tomasz Grzybowski, Grażyna Malukiewicz

**Affiliations:** ^1^Department of Ophthalmology, Faculty of Medicine, Nicolaus Copernicus University in Toruń, Collegium Medicum in Bydgoszcz, Sklodowskiej-Curie 9, 85-094 Bydgoszcz, Poland; ^2^Department of Molecular and Forensic Genetics, Institute of Forensic Medicine, Faculty of Medicine, Nicolaus Copernicus University in Toruń, Collegium Medicum in Bydgoszcz, Sklodowskiej-Curie 9, 85-094 Bydgoszcz, Poland; ^3^Clinic of Rheumatology and Connective Tissue Diseases, J. Biziel University Hospital No. 2, Bydgoszcz, Poland

## Abstract

**Purpose:**

To evaluate the expression profiles of the VEGF*α* and TGF*β* in the ERMs and ILMs in retinal disorders.

**Methods:**

In this nonrandomized prospective study, 75 patients (34 females and 41 males) referred to pars plana vitrectomy (PPV) due to different retinal diseases were enrolled to the study. The samples of ERMs and ILMs collected during PPV were immediately put in TRIzol® Reagent (Life Technologies, USA) and stored at −70°C until RNA extraction. Gene expression analysis was done with TaqMan® Gene Expression Assays (Applied Biosystems, USA) following the manufacturer's instructions.

**Results:**

The gene expression levels of VEGF*α* as well as of TGF*β*2 were significantly higher in ERMs than in ILMs in all studied groups. The level of TGF*β*2 expression exhibits a significantly lower values in iERMs as compared with the RRD group (*p* = 0.043). There were differences in TGF*β*2 expression in ILM in groups studied: DR versus RRD, *p* = 0.003; DR versus iERM, *p* = 0,047; and iERM versus RRD, *p* = 0.004.

**Conclusions:**

Our results revealed that factors associated with angiogenesis and wound healing processes in eyes with RRD, PDR, iERM, and MH were more upregulated in ERMs than in ILMs. This may indicate that ILM is not responsible for reproliferation and its peeling should be avoided in routine PPV.

## 1. Introduction

Proliferative vitreoretinopathy (PVR) is a severe retinal detachment (RD) and vitreoretinal surgery complication that can lead to severe vision reduction by tractional retinal detachment. Epiretinal membrane (ERM) is a semitransparent, membranous, pathologic tissue which grows on the internal surface of the retina at the vitreoretinal interface. ERMs can be either idiopathic or secondary to some pathologic conditions, including proliferative diabetic retinopathy, high myopia, uveitis, proliferative vitreoretinopathy, and other retinal degenerative diseases [1, 2]. Reported frequency of ERM falls between 3% to 8.5% after scleral buckling and 6.1% to 12.8% after vitrectomy [3–8].

Due to its structure, the ILM plays a key role in the development of the vitreous–retinal boundary. Composed of a basal membrane of Müller cells, proteoglycans, and type IV collagen fibers, it constitutes the so-called framework for the development of proliferative membranes. Müller cells are responsible for homeostatic and metabolic support of photoreceptors and neurons. They act as soft, compliant embedding for neurons, protecting them from mechanical trauma, and also release neuroactive signaling molecules that modulate neuronal activity [9]. The ILM peeling in the macula area allows removing all the ERMs and, as a result, all the tractions of the framework and the proliferation environment [10–12]. The majority of surgeons choose to peel the ILM away, but additional ILM peeling for ERM and RD surgery remains controversial. It is believed that ILM peeling during ERM removal may decrease the percentage of eyes experiencing its regrowth [13–15]. Nevertheless, vitreoretinal reproliferations are still among the chief causes behind unsuccessful vitreoretinal surgery. Moreover, ILM peeling has been known to cause mechanical traumatic changes to the retinal nerve fiber layer (RNFL) [16–20].

The molecular formation mechanism of both the primary and secondary ERMs in diabetic and nondiabetic patients is still poorly understood. A number of cytokines are involved in the ERM progression [21–23]. Recent technological advancements in genomics have given researchers new opportunities for identifying global gene expressions in specific tissues [24].

The aim of this prospective study was to analyse whether the gene expression profile of ERMs and ILM occurs in equal measure, which might aid in understanding ILM peeling.

## 2. Methods

Eighty-three patients (39 F, 44 M, age range: 26–84 years, mean age: 64.4, median: 66, standard deviation (SD): ±10.8), referred to the Department of Ophthalmology of the University Hospital in Bydgoszcz for 23-gauge pars plana vitrectomy (PPV) due to a variety of retinal diseases, were enrolled in the study. Prior to surgery, a detailed ophthalmic examination with OCT assessment was conducted, written informed consent was received from each patient before tissue sample acquisition, and approval for the study was granted by the ethics committee (KB 509/2013).

Three-port PPV with ERM removal and ILM peeling was the surgical technique used in this prospective study (DORC, the Polymed, Poland). Membrane removal and ILM peeling were performed with end-gripping forceps, intending to remove as much as possible of the ERM and remove the ILM from an area around the macula circa 3-4 disc diameter large. The surgeries were performed by two experienced vitreoretinal surgeons (JS and KK).

The patients enrolled in the study were categorized based on the disease they suffered from:

1. Idiopathic epiretinal membranes (iERMs)
2. Proliferative diabetic retinopathy (PDR)
3. Retinal detachment with epiretinal membranes (RD with ERMs)

As a control group, ILM samples collected during PPV performed due to MH from eight patients (5 females, 3 males) were used. Patient demographics are listed in Table 1.

ERM and ILM samples collected during PPV were promptly put in a TRIzol Reagent (Life Technologies, Foster City, CA, USA) and stored at −70°C until RNA extraction. RNA extracts were treated with DNase I using TURBO DNA-free™ Kit (Life Technologies, Foster City, CA, USA). Quantification of total RNA was performed by a DeNovix spectrophotometer (DeNovix, Wilmington, USA). Total RNA (800 ng) was reverse-transcribed using High-Capacity cDNA Reverse Transcription Kit (Life Technologies, Foster City, CA, USA) according to the protocol. Quantitative real-time reverse transcriptase PCR (qRT-PCR) was performed by TaqMan technology using a ViiA™ 7 Real-Time PCR System (Applied Biosystems, Foster City, CA, USA). Gene expression analysis was done with TaqMan Gene Expression Assays (Applied Biosystems, Foster City, CA, USA) following the manufacturer's instructions (Table 2). Negative control consisted of a PCR mix without cDNA. The glyceraldehyde-3-phosphate dehydrogenase (GAPDH) was used as the endogenous control to normalize gene expression levels for relative quantitative analysis through a comparative cycle threshold (ΔCt) method. Finally, the ΔΔCt method was used to compare gene expressions between ERM and ILM from PVR patients.

The data obtained is presented as a mean ± SD and median. For normally and equally distributed data, gene expression levels between the groups were compared using *t*-test. *p* < 0.05 was considered a statistically significant difference. Statistical analysis was performed using MS Excel 2010.

## 3. Results

### 3.1. Epiretinal Membrane

The gene expression levels of VEGF*α* as well as of TGF*β*2 were significantly higher in ERMs than in ILMs (*p* = 0.009 and *p* = 0.015, resp.; mean and SD values are presented in Table 3).

Comparison of gene expression of VEGF*α* and TGF*β*2 in ILM in patients who suffered from ERM to gene expression of VEGF*α* and TGF*β*2 in ILM in patients with MH (control group) reveals no statistical differences between these two groups.

### 3.2. Diabetic Retinopathy

The gene expression levels of VEGF*α* as well as TGF*β*2 were significantly higher in fibrous ERMs compared to ILMs (*p* = 0.016 and *p* = 0.003, resp., mean and SD values are presented in Table 4).

Comparison of gene expression of VEGF*α* and TGF*β*2 in ILM in patients suffered from PDR to gene expression of VEGF*α* and TGF*β*2 in ILM in the control group reveals a statistical difference between these two groups when it comes to TGF*β*2 but not VEGF*α* (*p* = 0.007 and *p* = 0.339, resp., mean and SD values are presented in Table 5).

### 3.3. Rhegmatogenous Retinal Detachment

The expression levels of VEGF*α* and TGF*β*2 were significantly higher in ERMs than in ILMs (*p* = 0.004 and *p* = 0.002, resp.; mean and SD values are presented in Table 6).

Comparison of gene expression of VEGF*α* and TGF*β*2 in ILM in patients suffering from RD to gene expression of VEGF*α* and TGF*β*2 in ILM in patients from the control group reveals a statistical difference between the two groups when it comes to TGF*β*2 but not VEGF*α* (*p* = 0.014 and *p* = 0.45, resp., mean and SD values are presented in Table 7), resembling the PDR group.

Comparison of gene expression of VEGF*α* and TGF*β*2 in ERMs between groups, depending on diagnosis, reveals no statistical differences between the groups when it comes to VEGF*α*. The level of TGF*β*2 expression exhibits significantly lower values in the iERM group compared to the RRD group (*p* = 0.043).

Similarly, comparison of gene expression of VEGF*α* and TGF*β*2 in ILMs between studied groups produces no observable statistical differences in term of VEGF*α*; however, such differences were found between all 3 studied groups when it comes to TGF*β*2 (DR versus RRD, *p* = 0.003; DR versus iERM, *p* = 0.047; and iERM versus RRD, *p* = 0.004). The RRD did exhibit the highest level and the iERM the lowest level of TGF*β*2.

## 4. Discussion

Roth and Foos postulated that an idiopathic ERM proliferates as retinal tissue-derived glial cells escape from microdefects in the internal limiting membrane (ILM) that occur during posterior vitreous detachment and migrate to the surface of the retina [25]. Another theory attributes the pathogenesis of an ERM to the growth and fibrous metaplasia of the vitreous cells that remain on the retina surface after posterior vitreous detachment. On the other hand, in the ERM that occur after rhegmatogenous RD, the retinal pigment epithelial cells are thought to migrate to the vitreous cavity through the retinal break and settle on the retinal surface, forming the membrane [26]. None of these theories put forth a reason behind certain patients developing PVR rapidly while others—not at all. The present study characterized the expression profiles of the inflammatory cytokines VEGF*α* and TGF*β*2 in the ERMs and ILMs in retinal disorders such as RRD, DR, and iERM. There might be several important factors that determine cytokine levels either inside the operated eye or the entire body. To overcome this problem, we collected both the ERM and ILM from the same eyes. Various cytokines, including the vascular VEGF, have been identified as playing a role in the pathogenesis of DR [27–29]. VEGF that was first discovered as a vascular permeability factor is specifically a mitogenic cytokine for vascular endothelial cells. Retinal ischemia is the basic stimulus leading to upregulation and increase of VEGF locally and therefore plays a major role in the progression of DR. Increased VEGF interacts with its two tyrosine kinase receptors on the retinal vasculature, resulting in the formation of new vessels and also disruption of the internal blood retinal barrier. The VEGF presence has been reported in iERMs [30, 31]. In previous studies, positive VEGF immunoreactivity of iERMs was found, an unsurprising fact considering that retinal glia have been known to produce VEGF. The question arose why no blood vessels in iERM were present despite the presence of VEGF. One possibility was the existence of other cells in the iERM besides endothelial cells that are targeted by VEGF. It was also plausible that the presence of endothelial growth inhibitory factors, such as TGF-*β*, may prevent VEGF from exerting its angiogenic activity. Nam et al. investigated the difference in the expression of specific growth factors (including VEGF and TGF *β*1) between diabetic and nondiabetic ERMs [32]. In our research, there were no statistical differences in VEGF*α* expression between the study groups either in ERM or ILM. There was a statistical difference (*p* = 0.043) in TGF*β*2 expression only between the RD group (mean = 5.60; SD = 2.46) and iERM group (mean = 4.16; SD = 3.36) with regard to ERM. Selim et al. observed that the elevation of VEGF levels was parallel to the severity of DR and to the degree of retinal ischemia, suggesting that the main pathogenic factor causing VEGF elevation and responsible for DR progression in their patients' eyes was retinal hypoxia [33]. Retinal diseases are closely associated with both decreased oxygenation and increased inflammation. It is unknown whether hypoxia-induced VEGF expression in the retina itself evokes inflammation, or whether inflammation is a prerequisite for the development of neovascularization. Interestingly, the majority of the previous studies evaluating the roles of different cytokines and growth factors in the pathogenesis of idiopathic epiretinal membrane were based on the vitreous samples. Few reports are related to expression levels of these factors between ERM and ILM and evaluated them not only in idiopathic epiretinal membranes, but also in fibrotic ones, usually seen in proliferative vitreoretinopathy, such as PDR or advanced RD. To address this issue, we assessed the gene expression levels of VEGF*α* and TGF*β*2 in idiopathic and fibrotic ERMs as well as in ILMs. To the best of our knowledge, this is the first study that compares these parameters in ERMs and ILMs in different diseases. Takahashi et al. showed significant differences in VEGF levels in vitreous body in RRD, MH, PDR, ERM, and RVO (*p* < 0.001) [34]. We found no statistical differences among RRD, PDR, and idiopathic ERM groups comparing VEGF*α* expression directly in ERMs; however, in RRD, we noticed the lowest expression. The evaluation of VEGF*α* expression directly in the ILM in RRD, PDR, and idiopathic ERM groups versus MH revealed no statistical differences contrary to Takahashi results. TGF*β*2 expressions showed statistical differences between almost all study groups, except one (ERM group versus MH group). Myojin et al. analysed gene expression in the irrigation solution collected during vitrectomy performed due to ERMs and MH and found that the expression levels of TGF*β*2 and VEGF*α* were significantly higher in eyes with iERM versus MH [35]. Nonetheless, we found no differences in gene expression of VEGF*α* and TGF*β*2 in ILM in patients suffering from ERM versus patients with MH. Similarly, there were no statistical differences in expression of VEGF*α* in ILM between RRD versus MH and DR versus MH groups. Interestingly, at the same time, there was a statistically higher expression of TGF*β*2 in ILM between RRD versus MH and DR versus MH groups. As it is well known, the transforming growth factor-*β* (TGF-*β*) and fibroblast growth factor (FGF) play crucial cooperative roles in fibrosis. For example, in the pathogenesis of proliferative vitreoretinopathy (PVR), TGF*β* plays a pivotal role, promoting transition of retinal pigment epithelial (RPE) cells into myofibroblasts. ERM is thought to be caused by a fibrocellular proliferation of the inner limiting membrane (ILM) and the subsequent vitreoretinal adhesion and traction [36, 37]. TGF*β* upregulation was reported in eyes with iERM, PDR, and PVR, and it is associated with intraocular fibrosis [21, 23, 30, 38]. Despite advances in surgical techniques, the percentage of unhealed PVR remains high, producing a failure rate of up to 10% in retinal surgical repairs [39]. In our study, TGF-*β* expression responsible for fibrosis was significantly higher in ERMs than in ILMs in all studied groups. One of the main targets of genetic studies is to translate evidence and benefits into clinical practice. ILM peeling is a subject of ongoing debate. Rinaldi et al. found no significant differences between postoperative best-corrected visual acuity or best-corrected visual acuity change in the ILM peeling group compared with the nonpeeling group. There was no significant difference in postoperative central macular thickness and central macular thickness reduction between the two groups [40]. Similarly, Díaz-Valverde et al. noticed that internal limiting membrane peeling does not improve the functional outcome after ERM surgery. The ILM peeling reduces ERM recurrences, but few recurrences are clinically significant [41]. Moreover, a number of researchers noticed that ILM peeling had been considered to cause mechanical retinal damage, including physiological alterations in Müller cells, irregularities of the nerve fiber layer, small paracentral scotomas, loss of Müller cells end-feet within the peeling area, and weakening of the macular glial structure [17, 42–44]. Müller cells react to mechanical and hypoxic stimuli by hypertrophy to resist and protect the neuroretinal layers from traction and to protect photoreceptors from apoptosis [45]. Gao et al. suggested that in some cases of myopic foveoschisis ILM removal resulted in the development of postoperative full-thickness macular holes [46]. Sakimoto et al. found by an image of en face OCT a retinal dimple signs after ILM peeling [47]. There are also results suggesting that ILM peeling may reduce retinal sensitivity and significantly increase the incidence of microscotomas [48]. A meta-analysis of vitrectomy with or without internal limiting membrane peeling for macular hole coexisting with retinal detachment in the highly myopic eyes done by Gao et al. revealed no definite benefit of postoperative visual improvement [49].

## 5. Conclusions

Our results reveal that VEGF*α* and TGF*β*2 associated with angiogenesis and wound healing processes in eyes with RRD, PDR, and iERM were more upregulated in ERMs than in ILMs. This may indicate that ILM is not responsible for reproliferation and its peeling should be avoided in routine PPV. Further studies are needed to better understand the ILM role in reproliferation and the need of ILM peeling and its consequences.

## Figures and Tables

**Table 1:** Demographics of patients included in the study groups.

	ERM group	DR group	RD group	Control group
Study population	16 (9 F/7 M)	21 (12 F/9 M)	38 (13 F/25 M)	8 (5 F/3 M)
Age (mean ± SD; min, max)	72 ± 6.53; min 61, max 82, med. 72	60.48 ± 9.06; min 54, max 73, med. 62	63.24 ± 10.54; min 26, max 82, med. 63.5	65.38 ± 15.94; min 30, max 79, med. 68

**Table 2:** The set of TaqMan Gene Expression Assays used for gene expression analyses.

Gene	Assay ID
GAPDH	Hs03929097_g1
VEGFA	Hs00900055_m1

**Table 3:** Expression levels of VEGF*α* and TGF*β*2 in the ERMs and ILMs in eyes with idiopathic epiretinal membranes (iERMs).

	ERM	ILM	*p* value
VEGF*α* (in iERM)			
Mean	4.43	2.55	**0.009**
Median	4.31	1.87	
Standard deviation	1.90	2.34	
TGF*β*2 (in iERM)			
Mean	4.16	1.66	**0.015**
Median	4.66	0.00	
Standard deviation	3.36	2.84	

**Table 4:** Expression levels of VEGF*α* and of TGF*β*2 in the fibrous epiretinal membranes (FERMs) and ILMs in eyes with proliferative diabetic retinopathy (PDR).

	FERM	ILM	*p* value
VEGF*α* (in PDR)			
Mean	4.34	2.96	**0.016**
Median	3.92	3.15	
Standard deviation	2.27	1.71	
TGF*β*2 (in PDR)			
Mean	5.53	3.23	**0.003**
Median	5.49	4.65	
Standard deviation	2.46	2.69	

**Table 5:** Comparison with the control group. Expression levels of VEGF*α* and of TGF*β*2 in the ILMs in the PDR group and control group.

	ILM study group	ILM control group	*p* value
VEGF*α* (in PDR)			
Mean	2.96	2.63	0.339
Median	3.15	2.65	
Standard deviation	1.71	2.46	
TGF*β*2 (in PDR)			
Mean	3.23	0.64	**0.007**
Median	4.65	0.00	
Standard deviation	2,69	1,92	

**Table 6:** Expression levels of VEGF*α* and of TGF*β*2 in the ERMs and ILMs in eyes with rhegmatogenous retinal detachment.

	ERM	ILM	*p* value
VEGF*α* (in RRD)			
Mean	3.71	2.53	**0.004**
Median	3.82	2.70	
Standard deviation	1.96	1.85	
TGF*β*2 (in RRD)			
Mean	5.60	14.92	**0.002**
Median	5.76	0.00	
Standard deviation	2.46	18.74	

**Table 7:** Comparison with the control group. Expression levels of VEGF*α* and of TGF*β*2 in the ILMs in the RRD group and control group.

	ILM study group	ILM control group	*p* value
VEGF*α* (in RRD)			
Mean	2.53	2.63	**0.45**
Median	2.70	2.65	
Standard deviation	1.85	2.46	
TGF*β*2 (in RRD)			
Mean	14.92	0.64	**0.014**
Median	0.00	0.00	
Standard deviation	18.74	1.92	
